# The Activation of GPR27 Increases Cytosolic L-Lactate in 3T3 Embryonic Cells and Astrocytes

**DOI:** 10.3390/cells11061009

**Published:** 2022-03-16

**Authors:** Dorian Dolanc, Tomaž M. Zorec, Zala Smole, Anja Maver, Anemari Horvat, Thanigaimalai Pillaiyar, Saša Trkov Bobnar, Nina Vardjan, Marko Kreft, Helena Haque Chowdhury, Robert Zorec

**Affiliations:** 1 Laboratory of Neuroendocrinology, Molecular Cell Physiology, Institute of Pathophysiology, Faculty of Medicine, University of Ljubljana, 1000 Ljubljana, Slovenia; dorian.dolanc@mf.uni-lj.si (D.D.); zala.smole@mf.uni-lj.si (Z.S.); anja.maver97@gmail.com (A.M.); anemari.horvat@mf.uni-lj.si (A.H.); nina.vardjan@mf.uni-lj.si (N.V.); helena.chowdhury@celica.si (H.H.C.); 2 Institute of Microbiology and Immunology, Faculty of Medicine, University of Ljubljana, 1000 Ljubljana, Slovenia; tomaz.zorec@celica.si (T.M.Z.); marko.kreft@celica.si (M.K.); 3 Laboratory of Cell Engineering, Celica Biomedical, 1000 Ljubljana, Slovenia; sasa.trkov@gmail.com; 4 Pharmaceutical/Medicinal Chemistry and Tübingen Center for Academic Drug Discovery, Institute of Pharmacy, Eberhard Karls University Tübingen, Auf der Morgenstelle 8, 72076 Tübingen, Germany; thanigaimalai.pillaiyar@uni-tuebingen.de; 5 Department of Biology, Biotechnical Faculty, University of Ljubljana, 1000 Ljubljana, Slovenia

**Keywords:** 3T3 embryonic cells, astrocytes, aerobic glycolysis, cytosolic L-lactate, FRET nanosensor, G-protein-coupled receptors, GPR27, agonists

## Abstract

G-protein-coupled receptors (GPCRs) represent a family with over 800 members in humans, and one-third of these are targets for approved drugs. A large number of GPCRs have unknown physiologic roles. Here, we investigated GPR27, an orphan GPCR belonging to the family of super conserved receptor expressed in the brain, with unknown functions. Cytosolic levels of L-lactate ([lactate]_i_), the end product of aerobic glycolysis, were measured with the Laconic fluorescence resonance energy transfer nanosensor. In single 3T3 wild-type (WT) embryonic cells, the application of 8535 (1 µM), a surrogate agonist known to activate GPR27, resulted in an increase in [lactate]_i_. Similarly, an increase was recorded in primary rat astrocytes, a type of neuroglial cell abundant in the brain, which contain glycogen and express enzymes of aerobic glycolysis. In CRISPR-Cas9 GPR27 knocked out 3T3 cells, the 8535-induced increase in [lactate]_i_ was reduced compared with WT controls. Transfection of the GPR27-carrying plasmid into the 3T3KOGPR27 cells rescued the 8535-induced increase in [lactate]_i_. These results indicate that stimulation of GPR27 enhances aerobic glycolysis and L-lactate production in 3T3 cells and astrocytes. Interestingly, in the absence of GPR27 in 3T3 cells, resting [lactate]_i_ was increased in comparison with controls, further supporting the view that GPR27 regulates L-lactate homeostasis.

## 1. Introduction

G-protein-coupled receptors (GPCRs), also known as seven-(pass)-transmembrane domain receptor proteins, form a large group of more than 800 evolutionarily related proteins in humans. These cell surface receptors detect molecules outside the cell and activate cellular responses. They represent important drug targets; approximately one-third of all approved drugs target members of this family [1,2]. GPCRs with unknown endogenous ligands are termed orphan; with more than 100 candidates, they represent new potential drug targets [2].

GPR27 is an orphan receptor, a member of the evolutionarily conserved family of GPCRs, termed super conserved receptor expressed in the brain (SREB) [3]. Their precise function is unknown; however, their localization in the brain, investigated by supersensitive in situ hybridization expression profiling, revealed their presence in most parts of the brain, including brain circuits that govern emotions and cognition [4]. By screening siRNAs targeting all GPCRs, GPR27 was identified as a novel regulator of insulin production [5], thus, playing a potential role in metabolism. To study the role of GPR27 in insulin secretion, a GPR27 knockout (KO) mouse was developed, however, with no gross abnormalities, although the weight of the animals was reduced, with slightly worsened glucose tolerance with lower plasma insulin levels while maintaining similar insulin tolerance [6].

In the central nervous system (CNS), astrocytes, an abundant type of neuroglia, are sensitive to insulin [7] and play key homeostatic roles in supporting neuronal networks [8]. Insulin, a hormone and a growth factor, acts on many cells, including adipocytes and skeletal muscle, to induce the incorporation of D-glucose transporters (i.e., GLUT4) into the plasma membrane, which facilitate the diffusion of D-glucose from the extracellular space into the cytoplasm, where D-glucose is degraded via the glycolytic pathway to pyruvate, which can be metabolized to L-lactate. Cytosolic pyruvate can also be transported into the mitochondrial matrix, converted to acetyl-CoA by the pyruvate dehydrogenase complex, and incorporated into the tricarboxylic acid cycle (TCA) [9]. Although neurons consume most of the energy in the CNS, they lack energy stores; these are present in the form of glycogen in astrocytes [10]. During enhanced neuronal activity, these stores can be used to produce L-lactate in astrocytes [11] and then be transferred to neurons to be used as fuel, a paradigm termed the astrocyte-to-neuron-lactate-shuttle (ANLS) [12] or discharged from the CNS [13]. The production of L-lactate in astrocytes occurs in the presence of oxygen, and this form of metabolism, termed aerobic glycolysis, also known as “the Warburg effect”, is characteristic of rapidly dividing cells, including cancer and embryonic cells, and in cells undergoing plastic morphological changes [14]. In astrocytes, this form of metabolism is operative in some CNS areas [15], as is the metabolic responsiveness to insulin by increasing the levels of glycogen [7]. Interestingly, GPR27 was proposed to mediate the glycerophospholipid plasmalogen-induced signal transduction of Akt and ERK [16], known metabolic intermediates activated through insulin. Therefore, here, we tested whether GPR27 may regulate aerobic glycolysis.

Two cell types were selected in this study that exhibit aerobic glycolysis: astrocytes [17] and 3T3-MEF murine embryonic cells (3T3 cells) with the genetic trait as seen in human ectodermal cancers [18]. The latter cells are ideal for genetic manipulation, and we generated CRISPR-Cas9 3T3 cell lines with selective KO of GPR27, in which we monitored the cytosolic level of L-lactate ([lactate]_i_) with a fluorescence resonance energy transfer (FRET) L-lactate nanosensor [19]. The results revealed that 8535 or 8535n, surrogate GPR27 agonists [20], increased [lactate]_i_ in wild-type (WT) 3T3 cells and astrocytes. However, 8535 failed to elicit an increase in [lactate]_i_ in the 3T3KOGPR27 cells. In a rescue experiment, where the plasmid encoding GRP27 was transfected into 3T3KOGPR27 cells, the results revealed a requirement for the GPR27 receptor. These results show that orphan receptor GPR27 regulates aerobic glycolysis by modulating [lactate]_i_ in 3T3 cells and in astrocytes.

## 2. Materials and Methods

### 2.1. Cell Cultures, Plasmid Transfection and Immunocytochemistry

3T3-MEF murine embryonic cells were kindly provided by Dr. K. Chylinski, Vienna Biocenter, University of Vienna, Vienna, Austria. The cells were grown in 25 mM D-glucose Dulbecco’s modified Eagle’s medium (DMEM), supplemented with 10% fetal bovine serum, 5 mM L-glutamine and 25 µg/mL penicillin/streptomycin in a vaporized atmosphere containing 95% air and 5% CO_2_ at 37 °C.

Primary astrocyte cultures were prepared from the cerebral cortices of 2- to 3-day-old female Wistar rats as described [17,21] and grown in high-glucose DMEM supplemented with 10% fetal bovine serum, 1 mM sodium pyruvate, 2 mM L-glutamine, 5 U/mL penicillin and 5 µg/mL streptomycin in a vaporized atmosphere containing 95% air and 5% CO_2_ at 37 °C until reaching between 70% and 80% confluence. Cell cultures were shaken overnight at 225 rpm, and the medium was changed the next morning; this was repeated three times. After the third overnight shaking, the cells were trypsinized and transferred to flat tissue culture tubes with a 10 cm^2^ growth area (Sarstedt Inc., Newton, NC, USA). This procedure yielded astrocytes with >95% purity, determined by anti-glial fibrillary acidic protein antibody staining [22,23].

The experimental animals were cared for in accordance with the International Guiding Principles for Biomedical Research Involving Animals developed by the Council for International Organizations of Medical Sciences and Animal Protection Act (Official Gazette of the RS, No. 38/13 and No. 92/20). The experimental protocol was approved by the Administration of the Republic of Slovenia for Food Safety, Veterinary and Plant Protection (Republic of Slovenia, Ministry of Agriculture, Forestry and Food), document no. U34401-26/2020/4. Every set of experiments was acquired from at least three different animals.

Before the experiments, cells were removed from the culture flasks with trypsin/EDTA and plated on 22-mm diameter glass cover slips (Sarstedt Inc.) coated with poly-L-lysine. After 24 h, cells were transfected with the plasmid construct Laconic [24] using Fugene 6 Transfection Reagent (Promega, Madison, WI, USA) or adenovirus-associated viral vector (Vector Biolabs, Malvern, PA, USA) according to the manufacturer’s instructions. Cells were exposed to the plasmids for 2 h and then further incubated for 24 h to allow the expression of the plasmid encoding proteins. The simultaneous co-transfection of cells with two plasmids: Laconic and a plasmid encoding GPR27 with the FLAG marker (GPR27-FLAG, a generous gift from Dr. Julien Hanson, University of Liège, Belgium, EU), were examined by immunocytochemical validation of successful transfection with anti-FLAG antibodies [20]. Briefly, the non-transfected 3T3KOGPR27 cells and the 3T3KOGPR27 cells expressing Laconic and/or GPR27-FLAG plasmid were washed with phosphate buffered saline (PBS) then permeabilized with ionomycin (4 µM) for 1–2 min before being washed with 3% bovine serum albumin (BSA) in PBS. The cells were then incubated with primary antibodies against FLAG tag (rabbit monoclonal, 1:600; Abcam, Cambridge, UK) for 10 min at room temperature. After washing with PBS, the cells were incubated for 20 min at room temperature with Alexa Fluor546-conjugated secondary antibodies (goat anti-rabbit polyclonal, 1:500, Molecular Probes by Life Technologies, Thermo Fischer Scientific, Waltham, MA, USA). Excess antibodies were washed off with PBS and the coverslips were mounted in a recording chamber on the microscope stage. Immunolabelled live cells were imaged with an inverted fluorescence microscope (Zeiss Axio Observer.A1, Zeiss, Oberkochen, Germany) with a Plan neofluar 20x/0.4 objective, Axiocam 702 camera and HXP 120 C Lamp Module (Zeiss, Oberkochen, Germany). Unless noted otherwise, all chemicals were purchased from Sigma-Aldrich (Merck KGaA, Darmstadt, Germany).

### 2.2. CRISPR-Cas9 Manipulation of 3T3 Cells

CRISPR-Cas9 manipulated 3T3 cells (Vienna Biocenter, Vienna, Austria) were prepared by retrieving GPR27 sequences from Ensembl and guide RNA (gRNA) targeting was designed using the CRISPOR tool (crispor.tefor.net (accessed on 26 April 2020)). gRNAs were selected primarily based on their specificity (at least three mismatches with at least one in the seed region to any off-target) and on predicted activity according to the Doench score. Targeting sequences were introduced into pX459 Cas9-p2A-puro plasmid (Addgene) via BbsI cloning. Plasmids (3 µg) were introduced into mouse embryonic stem cells (ESCs, 1 × 10^6^) by electroporation with a Neon electroporator (Thermo Fisher Scientific, Waltham, MA, USA) according to the manufacturer’s protocol. Cells were selected 24 h after electroporation with puromycin (0.5 µg/mL) and collected and lysed for genotyping 72 h later. Polymerase chain reaction (PCR) products were column purified (GeneJET Gel Extraction Kit) and sent for sequencing with primer CR3218 (GCCTGCCCGCAGTGAA). Editing efficiency was analyzed with the TIDE algorithm (https://tide.deskgen.com/ (accessed on 6 May 2019)) based on chromatogram analysis with the WT ESC PCR product used as a reference. Three targeting sequences were cloned into an in-house template vector p31 containing the T7 promoter followed by BbsI cloning sites, the optimized gRNA scaffold and the DraI restriction site used for template linearization. The resulting gRNA transcription was performed with a HiScribe T7 High Yield RNA Synthesis Kit (New England Biolabs, Ipswitch, MA, USA) according to the manufacturer’s protocol, and gRNA was purified via extraction with phenol/chloroform/isoamyl alcohol (25:24:1; Applichem, Darmstadt, Germany) followed by ethanol precipitation. The concentration of RNA was measured with Nanodrop. Concentration and RNA integrity were verified by denaturing gel electrophoresis (10% polyacrylamide/urea/1 × TAE) and UV shadowing. Cells reaching 70–80% confluence were trypsinized and counted. One million living cells were used for electroporation with 12 µg of gRNA pre-mixed with 5 µg of Cas9 protein in Cas9 buffer (20 mM HEPES [pH 7.5], 150 mM KCl, 0.5 mM DTT, 0.1 mM EDTA). Electroporation was performed using a Neon Transfection System with 100-µL neon pipette tips using the recommended electroporation protocol (1350 V pulse; 30 ms pulse width, 1 pulse). Electroporated cells were cultured in DMEM supplemented with 10% FCS and L-Gln. Normocin (antibiotic/antimycotic; InvivoGen, San Diego, CA, USA) was added after approximately 2 h.

Part of the batch culture was collected 24 h after electroporation for genotyping to confirm editing. Single clones of 3T3 cells electroporated with g1471 were prepared in 96-well plates by serial dilution of the culture calculated to 0.5 cell per well. Clones were cultured in medium supplemented with 50% conditioned medium (1-day-old medium collected from actively growing cells) for 3 weeks with weekly media exchange. Confluent clones were collected for PCR genotyping as described above. PCR bands suggesting large changes on different alleles were cut out of the gel, purified with the GeneJET Gel Extraction Kit and sent for sequencing separately. Six putative KO clones with following TIDE-determined genotypes were expanded. New cell lysates were prepared and the genotyping PCR was repeated. To confirm the genotype, the PCR products were cloned into an in-house blunt-end cloning vector and sequenced (Figure S1). After verification, three clones were selected as putative KO cells.

### 2.3. FRET Measurements of Cytosolic L-Lactate Levels

3T3 cells expressing the FRET-based L-lactate nanosensor Laconic were examined between 24 and 48 h after the transfection with a Zeiss Axio Obsever.A1 (Zeiss, Oberkochen, Germany) fluorescence microscope with a CCD camera and monochromator (Polychrome V; Till Photonics, Graefelfing, Germany) with a monochromatic light source at a wavelength of 436 nm. Dual emission intensity ratios were recorded using an image splitter (Photometrics DV2, Tucson, AZ, USA) and two emissions filters (465 nm for cyan fluorescence protein (mTFP), 535 nm for yellow fluorescence protein (Venus)). Images were acquired every 10 s with an exposure time between 0.1 and 0.8 s at room temperature (23 °C–25 °C). Astrocytes expressing Laconic were examined by confocal microscopy (Zeiss LSM 780 laser scanning microscope, Zeiss, Oberkochen, Germany). Cells were excited at 458 nm, and fluorescent emissions of the donor (mTFP) and acceptor (Venus) were collected every 10 s at room temperature (23 °C–25 °C) from 473–499 nm and 508–534 nm, respectively.

Coverslips with transfected cells were mounted in a recording chamber on the microscope stage. Initially, these cells were kept in standard extracellular solution (10 mM HEPES/NaOH, 0.5 mM NaH_2_PO_4_·H_2_O, 5 mM NaHCO_3_, 135.3 mM NaCl, 5 mM KCl, 1.8 mM CaCl_2_, 2 mM MgCl_2_, 3 mM D-glucose; pH 7.4, ~300 mOsm osmolality) and then after 290–300 s (baseline), treated with various stimuli: 8535 (N-[4-(anilinocarbonyl)phenyl]-2,4-dichlorobenzamide (Tocris, Bristol, UK) and 8535n (N-[4-(anilinosulfonyl)phenyl]-2,4-dichlorobenzamide), both at 1 µM, GPR27 surrogate agonists [20]; ChemBridge Corporation, San Diego, CA, USA) and diluted in DMSO (final concentration 0.01%). The 8535n was synthesized as in [25] of sufficient purity (Figure S2). In the control experiments, only the vehicle (standard extracellular solution) in which the compounds were dissolved was applied. In all experiments, between 10 and 20 mM L-lactate (sodium L-lactate; Sigma) was applied at the end of the recording as a positive control for the responsiveness of the Laconic nanosensor.

**Figure 1:**
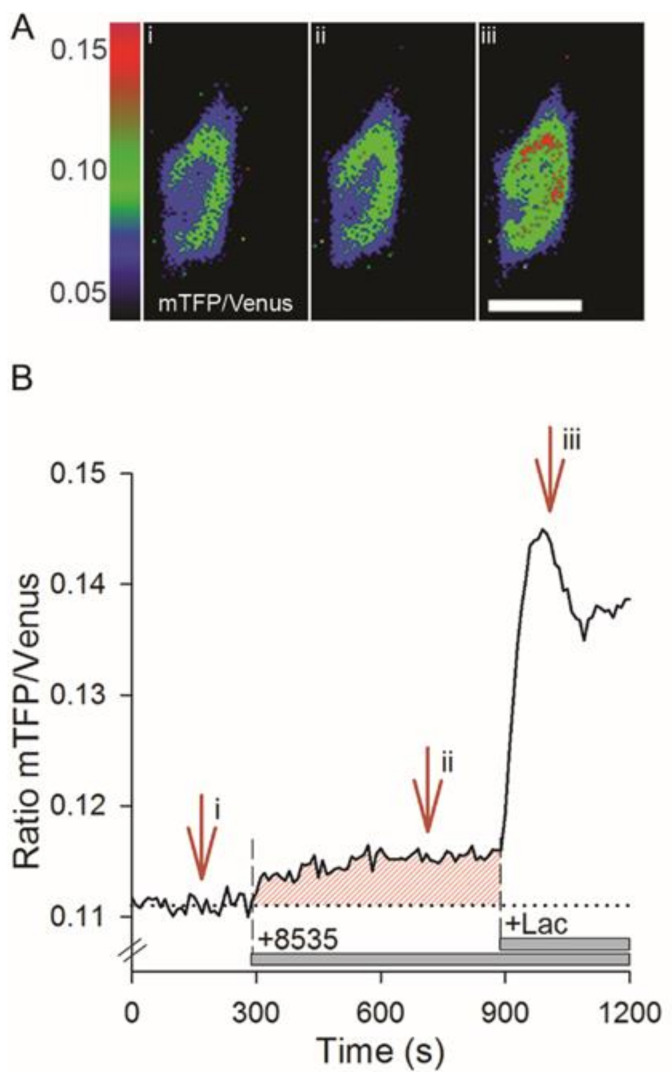
Effect of 3T3 cell stimulation with 8535, a surrogate agonist of GPR27 on [lactate]_i_. (**A**) Pseudo-colored micrographs display changes in the mTFP/Venus ratio of the lactate sensor Laconic in a single 3T3 cell, reporting cytosolic levels of L-lactate ([lactate]_i_ (FRET ratio, see Methods). Laconic expression is predominantly present in the cytoplasm. Representative images display a 3T3 cell at the beginning of experiment (**i**) and after application of 1 µM 8535 (**ii**) and 20 mM L-lactate (**iii**). Extracellular L-lactate was applied as a positive control to test if the Laconic sensor is functionally expressed in the 3T3 cells (i.e., responds to intracellular L-lactate elevations). Scale bar, 20 µm. (**B**) The mTFP/Venus ratio acquired from a time series of micrographs of the cytoplasmic region of the cell in (**A**). Note the increase in the signal after the application of 8535 and extracellular L-lactate, 20 mM (+Lac; horizontal bar). The arrows denote the frames shown in (**A**). The red-striped area denotes the area under the curve.

The FRET ratio signal was obtained by the integration of the region of interest selecting the whole cell using Life Acquisition software (Till Photonics, Graefelfing, Germany) and Zeiss Zen Imaging software (Carl Zeiss Microscopy, Jena, Germany), respectively. As a FRET pair, Laconic uses mTFP and Venus [19]. The background fluorescence was subtracted from individual mTFP and Venus fluorescence signals. Normalization to the baseline was used to determine agonist-induced changes in the FRET ratio. Changes in the FRET ratio signal were calculated and analyzed using Microsoft Excel (Microsoft, Redmond, WA, USA). An increase in the normalized FRET ratio indicates an increase in [lactate]_i_ [17]_._ The cells were defined as responsive when the average change in the FRET signal (∆FRET), determined after the addition of the stimulus from 320–420 s and from 300–690 s after the start of recording in 3T3 cells and astrocytes, respectively, was higher than three standard deviations of the averaged baseline value (time interval, 190–290 s, just before the addition of agonists). Other cells were defined as unresponsive. The area under the curve (AUC, see Figure 1) was calculated for each normalized response curve within the time interval from the addition of the stimulus to the addition of between 10 and 20 mM L-lactate, a positive control. The average AUC was calculated for each stimulus and the corresponding controls. The moving average with an interval of 5 points was used to smooth out short-term signal fluctuations of the signals.

### 2.4. Statistical Analysis

Data are presented as means ± SEM or medians with interquartile range (boxplots). Normal distribution was tested using the Shapiro–Wilk test, and probability distribution was tested with the Kolmogorov–Smirnov test. The statistical significance between two groups was determined with a Student’s *t*-test or Mann–Whitney rank-sum test, as appropriate. The statistical significance between more than two groups was determined with one-way or two-way analysis of variance [26] on ranks. All statistical analyses were conducted using SigmaPlot software (SyStat, San Jose, CA, USA). A two-tailed *p* value ≤ 0.05 was considered to be significant.

## 3. Results

### 3.1. A Surrogate Agonist of GPR27 Increases [lactate]i in 3T3 Cells

Previously, it was shown that the application of an agonist (L-lactate or 3-chloro-5-hydroxybenzoic acid), selective for the canonical L-lactate receptor GPR81, induces an adenylate cyclase-dependent increase in cytosolic L-lactate ([lactate]_i_) in astrocytes and in 3T3 cells, confirming the presence of aerobic glycolysis in these cells [17]. Here, we tested whether the application of 8535, a surrogate agonist of GPR27 [20], also elicits an increase in [lactate]_i_. Figure 1A depicts representative images of 3T3 cells expressing Laconic [19]; levels of [lactate]_i_ are represented by the color-coded mTFP/Venus ratio FRET signal, reporting [L-lactate]_i_. Figure 1B shows the time-dependent response recorded in the cell depicted in Figure 1A, where the application of 8535 (1 µM) is followed by L-lactate (20 mM), a positive control to validate that Laconic-expressing cells respond to increased extracellular L-lactate. The time-dependent increase in [lactate]_i_ was characterized by an exponential increase to a steady state, as reported previously for agonist-induced increases in 3T3 cells and astrocytes [17]. To quantify these responses, we integrated the area under the curve (AUC), relative to the basal level (in %), as depicted in Figure 1B. The percentage of cells responsive to 8535 application was 33% (13 of 39 cells tested). The average AUC of 8535-induced increase in [lactate]_i_ was 855.2 ± 185.3% in responsive cells and 314.5 ± 90.7% in all cells tested, both significantly higher than the controls (*p* < 0.001; Table 1).

These results indicate that 8535, a surrogate agonist for GPR27 [20] increases aerobic glycolysis in 3T3 cells, measured as increases in [lactate]_i_.

### 3.2. Reduced 8535-Induced Changes in [lactate]_i_ in 3T3KOGPR27 Cells Are Rescued by Transfection of Plasmid Encoding GRP27

To determine whether responses to 8535 recorded in Figure 1 require the expression of GPR27, CRISPR-Cas9 3T3 cell lines were generated (see Methods) by knocking out the orphan-designated GPR27. If this gene mediates the response in [lactate]_i_ (Figure 1), we reasoned that stimulation of 3T3KOGPR27 cells by 8535 (1 µM) should exhibit a reduced change in [lactate]_i_. Furthermore, if GPR27 mediates the increase in [lactate]_i_ then transfecting the plasmid encoding GPR27 should be able to reverse the lack of [lactate]_i_ responses in 3T3KOGPR27 cells.

**Figure 2:**
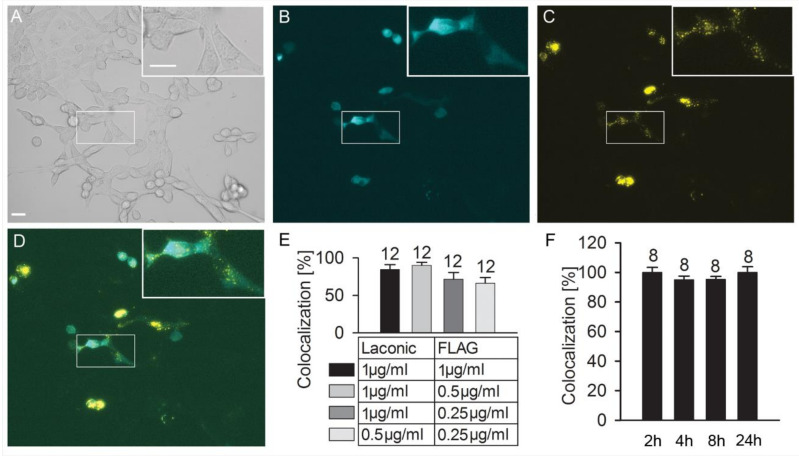
Optimization of co-transfection of plasmids Laconic and GPR27-FLAG into 3T3 cells. Immunocytochemistry of 3T3KOGPR27 cells co-transfected with Laconic and GPR27-FLAG (FLAG) plasmids and labeled with anti-FLAG antibody (see Methods). (**A**) Cells recorded under a Zeiss HAL 100 light source. Scale bars, 20 μm. (**B**) Cells expressing plasmid Laconic. (**C**) Cells expressing plasmid GPR27-FLAG and stained with anti-FLAG antibodies. (**D**) Merged images of Laconic- and GPR27-FLAG-positive signals. Pixel value ranges from 0 to 255. (**E**) Co-localization of the Laconic and GPR27-FLAG plasmids, depending on different plasmid concentrations. (**F**) Co-localization of Laconic and GPR27-FLAG depending on different plasmid incubation times. The numbers adjacent to the bars are the number of cells analyzed.

**Figure 3:**
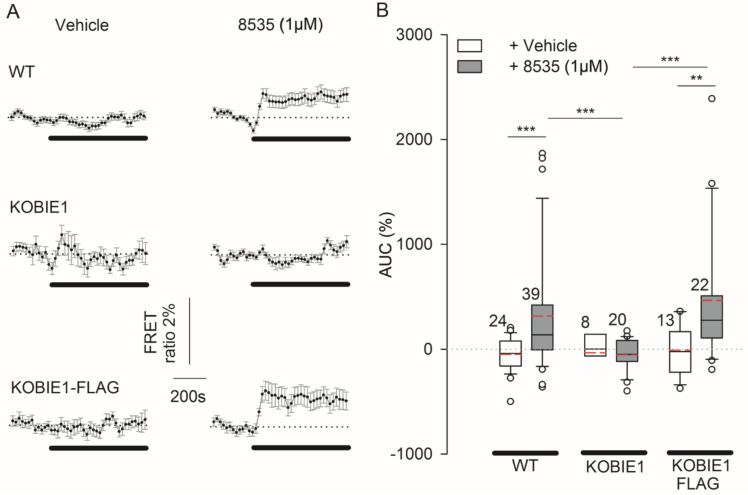
Surrogate GPR27 agonist 8535-induced increase in [lactate]_i_ in GPR27 knockout 3T3 cells is rescued by cell transfection with GPR27 plasmid. (**A**) Mean normalized time course of the Laconic FRET ratio signals reporting [lactate]_i_, on the addition of extracellular solution (vehicle, left columns) and agonist 8535 (1 µM, right columns) in 3T3WT cells (WT), in cells with knocked out GPR27 (KOBIE1) and in KOBIE1 cells with overexpressed GPR27 (KOBIE1FLAG). Horizontal black lines indicate the presence of stimuli; the horizontal dotted lines represent the relative baseline value of 1. (**B**) Boxplot comparison of the median area under the curve (AUC) of the Laconic FRET ratio signal, relative to basal levels (in %), on the addition of extracellular solution (vehicle, white) and agonist 8535 (1 µM; gray) in WT, KOBIE1 and KOBIE1-FLAG cells. Red dashed lines in the boxes represent the average values; white circles are outliers. Two-way and one-way ANOVA on ranks were used. Mann-Whitney rank sum tests were used for the comparison of isolated groups (** *p* ≤ 0.01, *** *p* ≤ 0.001). Numbers adjacent to the boxes are the number of cells analyzed from at least three independent cell passages.

To carry out the latter experiments, we optimized the conditions for the simultaneous co-transfection of cells with two plasmids: Laconic and a plasmid encoding GPR27 with the FLAG marker (GPR27-FLAG), allowing immunocytochemical validation of successful transfection with anti-FLAG antibodies [20]. Figure 2 displays representative images of 3T3 cells co-expressing Laconic (Figure 2B) visualized through its fluorescence, and GPR27-FLAG (Figure 2C) visualized through anti-FLAG antibodies fluorescence. Figure 2D shows the mask of the two respective fluorescence signals. The optimization of the co-transfection was conducted by testing different concentrations of both plasmids (Figure 2E) and different plasmid incubation times (Figure 2F). The highest co-localization between GPR27-FLAG and Laconic signals was 90.1% ± 3.9% after transfecting the cells with 0.5 µg/mL and 1 µg/mL of the plasmids, respectively (Figure 2E). The exposure of the cells to the plasmid for 2 h, washed and then incubated further for 24 h yielded the highest co-localizations of the two plasmid products, 100% ± 3.4% and 100% ± 3.9%, respectively (Figure 2F). This protocol preserved the relatively high viability of cells, hence, we used it in our experiments. These parameters were used in the experiments depicted in Figure 3.

The results in Figure 3A display averaged traces of a Laconic FRET ratio signal, recorded in WT and in 3T3KOGPR27 cells in the control experiments (vehicle added) and when 8535 (1 µM) was applied. The stimulation of 3T3WT cells with 8535 (1 µM) resulted in an increase in [lactate]_i_ with an average AUC of 314.5 ± 90.7% (*n* = 39), significantly higher than the AUC in vehicle-stimulated cells (−50.4 ± 32.9%; *n* = 24; *p* ≤ 0.001; Figure 3B). As expected, in 3T3KOGPR27 cells, the responses were reduced to −50.9 ± 32.7% (*n* = 20), not different from vehicle-stimulated cells (−34.6 ± 87.8%; *n* = 8), yet significantly lower than for 3T3WT cells (*p* = 0.001; Figure 3B). However, in 3T3KOGPR27 cells pre-transfected with the GPR27FLAG plasmid (KOBIE1-FLAG), the 8535-induced responses increased significantly in comparison to the response in 3T3KOGPR27 cells (464.2 ± 134.5%; *n* = 22; *p* ≤ 0.001; Figure 3B) and were also significantly higher than those in vehicle-stimulated KOBIE1-FLAG cells (−10.5 ± 65.6%; *n* = 13; *p* ≤ 0.01).

These results further demonstrate that the GPR27 receptor regulates [lactate]_i_ in 3T3 cells.

**Figure 4:**
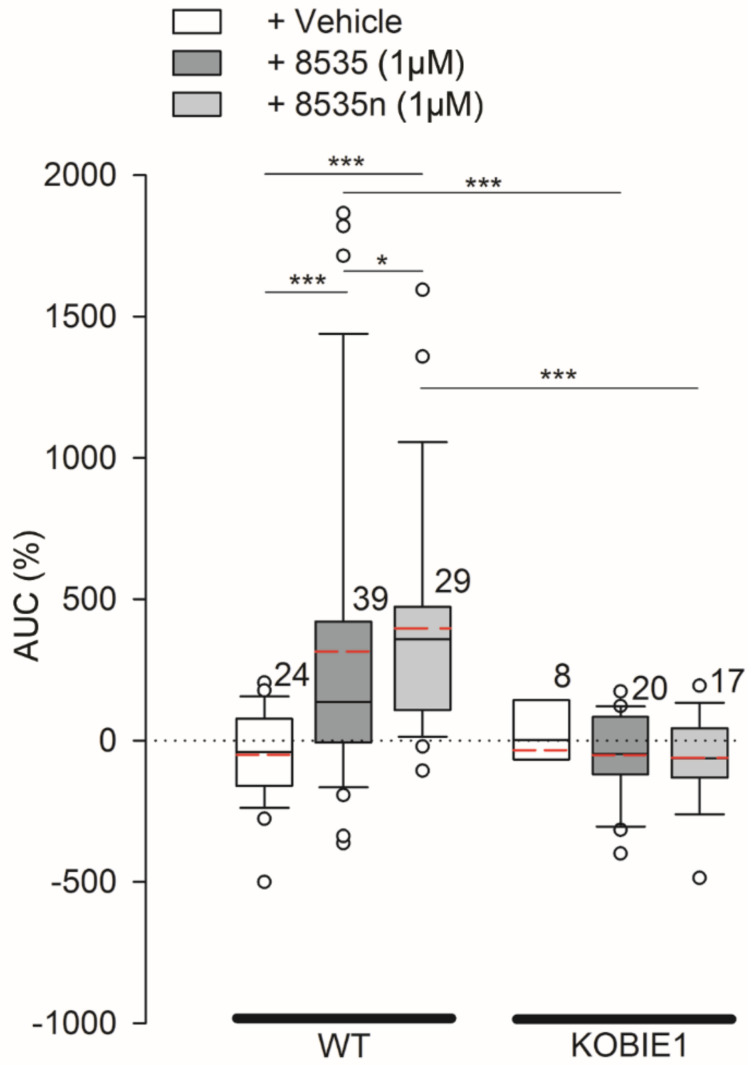
Comparison of responses in [lactate]_i_ on stimulation with 1 µM 8535 and 8535n, surrogate agonists of GPR27 in 3T3 cells. Comparison of the median area under the curve (AUC, %) of the Laconic FRET ratio signal on the addition of extracellular solution (vehicle, white boxes), GPR27 agonist 8535 (1 µM; dark gray boxes), and GPR27 agonist 8535n (1 µM; light gray boxes), in 3T3WT (WT) and 3T3KOGPR27 (KOBIE1) cells. For the difference between 8535 and 8535n (see Methods). Red dashed lines in boxes represent the average values, white circles are outliers. Note that the 8535n elicits larger AUC in 3T3 cells than 8535. Two-way and one-way ANOVA on ranks were used. Mann–Whitney rank sum tests were used for the comparison of isolated groups (* *p* ≤ 0.05, *** *p* ≤ 0.001). Numbers adjacent to boxes are the number of cells analyzed from at least three independent cell passages.

To further validate this claim, similar data were obtained with another surrogate GPR27 agonist described previously [20], referred to here as 8535n (1 µM; Figure 4). Although the chemical structure of the two surrogate agonists is similar [20] (see also Methods), the average AUC induced in 3T3WT cells by 8535n was 490.03 ± 87.95% (*n* = 29), significantly higher (*p* < 0.05) than that recorded with 8535 (314.5 ± 90.7%; *n* = 39; Figure 4). In 3T3KOGPR27 cells, stimulation with either agonist did not elicit responses different from the controls. Taken together, these data confirm that surrogate selective agonists for GPR27 (8535 and 8535n) require the expression of GPR27 to elicit an increase in [lactate]_i_ in 3T3WT cells and that GPR27 activation regulates aerobic glycolysis. We further investigated whether GPR27 is also active in modulating resting [lactate]_i_, without any stimulation.

### 3.3. Resting [lactate]_i_ Is Increased in 3T3KOGPR27 Cells in Comparison with 3T3WT Control Cells

**Figure 5:**
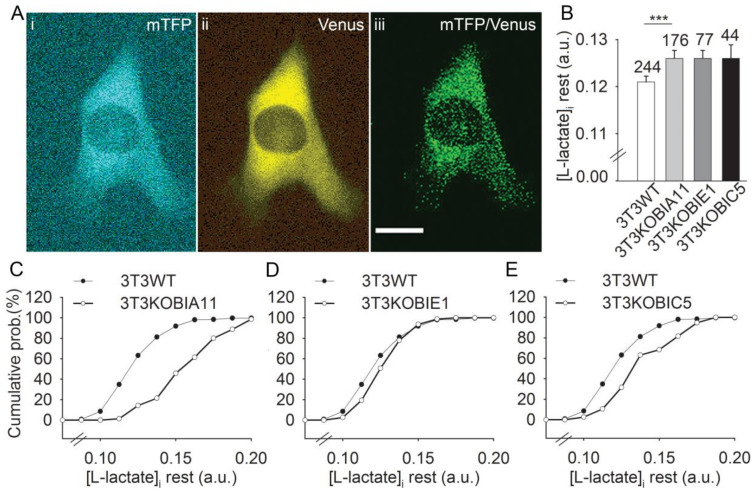
Resting levels of [lactate]i in 3T3GPR27KO cells (3T3KOBIA11, 3T3KOBIE1 and 3T3KOBIC5) are increased in comparison with 3T3WT. (**A**) Fluorescent micrographs display resting 3T3 cells expressing the lactate nanosensor Laconic: (**i**) mTFP channel visible in cyan, (**ii**) Venus channel visible in yellow and (**iii**) mTFP/Venus ratio, a parameter related to levels in [lactate]_i_; the lowest mTFP/Venus ratio in the background is represented as black pixels; green pixels represent mTFP/Venus ratio > 0. For calculation of the average cell resting levels of [lactate]_i_, the region of interest included the whole cell image area. Scale bar, 20 µm. (**B**) Average resting [lactate]i levels measured with the Laconic FRET ratio signal (mTFP/Venus) revealed that in 3T3KOBIA11 cells, resting [L-lactate]i is significantly increased (one-way ANOVA on ranks and Mann–Whitney rank sum test for the comparison of isolated groups; *** *p* ≤ 0.001). Numbers adjacent to bars are the number of cells analyzed. The distribution graphs show the comparison of the cumulative frequency distribution of the average basal FRET ratio signals in arbitrary units (a.u.) representing resting [L-lactate]i in single 3T3 wild-type cells (WT, black circles) and in (**C**) 3T3KOBIA11, (**D**) 3T3KOBIE1, (**E**) 3T3KOBIC5 cells (white circles). The resting value frequency distributions of the three 3T3KOB cells were tested for equality against the resting value distributions of the 3T3WT cells with the two-sample Kolmogorov–Smirnov test. The frequency distributions obtained in 3T3KOBIA11 and 3T3KOBIC5 cells differ significantly from the 3T3WT distribution (D_(3T3KOBIA11)_ = 0.597 > 0.134; D_(3T3KOBIC5)_ = 0.315 > 0.222); the frequency distribution in 3T3KOBIE1 cells is not different from controls.

To learn whether GPR27 affects resting [lactate]_i_, we monitored the FRET ratio (mTFP/Venus) in 3T3WT and in the three 3T3KOGPR27 cell lines we generated (KOBIA11, KOBIC5 and KOBIE1). Figure 5 displays fluorescent micrographs of a resting 3T3WT cell expressing the Laconic nanosensor: mTFP channel in cyan (Figure 5A), Venus channel in yellow (Figure 5B) and the mTFP/Venus ratio in green, a parameter related to [lactate]_i_ (Figure 5C). In all three 3T3KOGPR27 cell lines tested, the average resting level of [lactate]_i_ appeared increased in comparison with the control. Although the increase was statistically significant in the KOBIA11 cells (*p* < 0.001; ANOVA; Figure 5D), the average [lactate]_i_ in KOBIC5 and KOBIE1 cells was not different to the controls. However, we also tested the equality of the frequency distribution of the FRET ratio resting values between the tested 3T3KOGPR27 cells and the 3T3WT controls, revealing that the frequency distributions of KOBIA11 and KOBIC5 cells were significantly different compared with the 3T3WT cells (two-sample Kolmogorov–Smirnov test; Figure 5) and the frequency distribution of FRET resting values in KOBIE1 cells did not differ from that in the 3T3WT controls. These results indicate that at rest, GPR27 may play a constitutive role in regulating L-lactate homeostasis.

### 3.4. Increased [lactate]_i_ in Astrocytes Stimulated by Surrogate Agonists of GPR27

**Figure 6:**
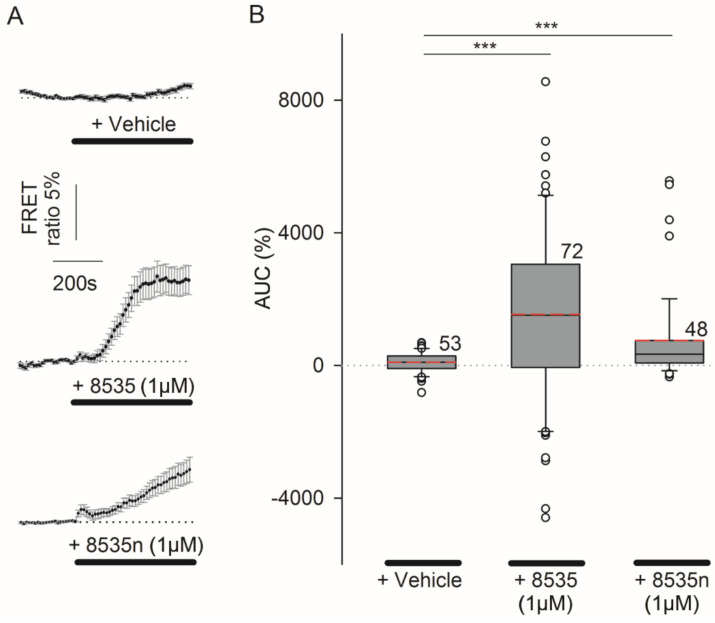
The GPR27 surrogate agonist 8535-induced increase in [lactate]_i_ in astrocytes. Astrocytes were stimulated with 8535 or 8535n, surrogate agonists of GPR27, and cytosolic lactate ([lactate]_i_) was measured by the FRET-based nanosensor Laconic. (**A**) Average normalized time course of the Laconic FRET ratio signal (mTFP/Venus) with the addition of vehicle (**top** panel), 8535 (1 µM, **middle** panel) or 8535n (1 µM, **lower** panel). Horizontal black lines indicate the presence of stimuli; the horizontal dotted lines represent the relative baseline value of 1. (**B**) Boxplots compare the median values of the area under the curve (AUC, %) of the Laconic FRET ratio signal on the addition of vehicle, 8535 (1 µM) and 8535n (1 µM). Red dashed lines in the boxes represent the average values; white circles are outliers. One-way ANOVA on ranks was used. Mann–Whitney rank sum tests were used for the comparison of isolated groups (*** *p* ≤ 0.001). Numbers adjacent to boxes are the number of cells analyzed and prepared from at least three animals.

Like 3T3 cells, astrocytes also exhibit aerobic glycolysis [17,27,28]. Therefore, we nex*t*-tested whether the pharmacologic activation of GPR27 may increase [lactate]_i_ in astrocytes. Both surrogate agonists of GPR27, 8535 and 8535n, were used to stimulate astrocytes and [lactate]_i_ was measured with the FRET-based nanosensor Laconic. Figure 6A displays average normalized time courses of the Laconic FRET ratio signal (mTFP/Venus) with the addition of vehicle (top panel), 8535 (1 µM, middle panel) and 8535n (1 µM, lower panel). The average 8535-induced increase in AUC was 1515.4 ± 306.9% (*n* = 71), significantly higher than the 8535n-induced increase in [lactate]_i._ of 749.3 ± 193.4%·s (*n* = 48; *p* ≤ 0.05). However, both ligands induced a significantly higher average increase in [lactate]_i_ than the vehicle (93.8 ± 42.3%, *n* = 53, *p* ≤ 0.001, one-way ANOVA on ranks and Mann–Whitney rank sum tests for the comparison of isolated groups). Boxplots (Figure 6B) compare the median values of AUC (%·s) of the Laconic FRET ratio signal with the addition of 8535 (1 µM), 8535n (1 µM), and the control. The responsiveness to 8535 application was 59% (42 of 71 cells tested) and responsiveness to 8535n application was 54% (26 of 48 cells). In the control cells, an apparent response to stimuli was detected in 20% (11 of 53) of the cells, likely due to the mechanical sensitivity of astrocytes [29] because the addition of a bolus solution may result in mechanical stimuli. Note that in responsive cells, the average AUC differed if cells were stimulated by 8535 or 8535n (Table 1). The average 8535-induced increase in AUC was 3136.9 ± 283.5% and the average 8535n-induced increase was 1330.4 ± 313.0%, both significantly higher than in the responsive control cells (392.9 ± 55.4%; *p* < 0.001 and *p* < 0.01; Table 1).

These results revealed that in astrocytes, GPR27 agonists elicit an increase in [lactate]_i_ similar to in 3T3 cells, supporting the view that in astrocytes, GPR27 activation enhances aerobic glycolysis (Figure 6).

## 4. Discussion

Here, we investigated the involvement of the GPR27 orphan receptor in the regulation of the intracellular levels of L-lactate, the end product of aerobic glycolysis. Despite the presence of adequate levels of oxygen, this form of metabolism is characteristic for cancer and embryonic stem cells, and in cells with dynamic morphological plasticity [14], including astrocytes [23,30]. Although aerobic glycolysis is not very efficient in producing ATP, it generates biosynthetic intermediates, which are an essential advantage for developing and growing tissues [31].

The experiments in this study revealed that pharmacologic activation of GPR27 in 3T3 embryonic cells and astrocytes increases [lactate]_i_. The endogenous ligand of GPR27 is unknown, but surrogate selective agonists, including the 8535 and 8535n molecules (see Methods), have been described [20], allowing further studies of the function of this receptor, as used in this study.

The results of this study clearly demonstrate that the GPR27 orphan receptor plays a role in the regulation of aerobic glycolysis; however, the exact cytosolic signaling leading to a GPR27-mediated increase in [lactate]_i_ will need to be investigated in further studies. Three lines of evidence obtained in this study support the role of GPR27 in the regulation of aerobic glycolysis. First, in the absence of GPR27, pharmacologic activation with the application of 8535 failed to elicit an increase in [lactate]_i_ in 3T3 cells (Figure 3). Second, the responses in [lactate]_i_ were rescued by transfecting a plasmid encoding GPR27 into the GPR27 knocked-out 3T3 cells (Figure 3). Third, the resting [lactate]_i_ levels were shown to increase in GPR27-defficient cells (Figure 5), indicating that L-lactate production at rest may be regulated by GPR27. Moreover, surrogate GPR27 selective agonists, 8535 and 8535n, also elicited an increase in [lactate]_i_ in astrocytes (Figure 6), the key homeostasis-providing cells in the CNS [32]. In these cells, aerobic glycolysis is operative during neurodevelopment and even in adulthood in some areas of the CNS [15]. The production of L-lactate is regulated by adrenergic and many other GPCR receptors [28,33,34,35]; this study shows that GPR27 may also play a role.

Astrocytes were more likely to respond to stimuli with GPR27 agonists compared with 3T3 cells, thus, it could be assumed that GPR27 is expressed more abundantly in astrocytes than in 3T3 cells. On the other hand, astrocytes are also sensitive to mechanical stimulation [29], which may explain relatively high apparent responsiveness to a bolus application of vehicle in the control experiments (Figure 6, Table 1). Nevertheless, the increase in AUC, a measure of increased [lactate]_i_, appears to be much greater in astrocytes compared with 3T3 cells. There are also some differences between the two agonists in terms of the response they elicit. In astrocytes, 8535 appears to be a much stronger agonist than 8535n, eliciting a more rapid and higher increase in [lactate]_I_, although both are considered specific surrogate agonists for GPR27 [20]. In contrast, in 3T3WT cells, these agonists seem similarly potent (Figure 4).

It was shown recently that the noradrenaline-stimulated production of L-lactate in astrocytes requires the entry of D-glucose and transit through the glycogen shunt [11] and that this is regulated by Ca^2+^ and cAMP second messengers [27]. It will be interesting to learn whether the activation of GPR27 is associated with increased glycogenolysis, as is the case with the activation of adrenergic receptors in astrocytes [13], and whether this depends on cytosolic Ca^2+^ and/or cAMP second messengers [17,27]. Moreover, stressed astrocytes accumulate lipid droplets [36], and whether GPR27 activation is involved in lipid droplet metabolism warrants further studies.

In summary, this study provides evidence that GPR27 plays a role in aerobic glycolysis because the activation of cells by a selective GPR27 agonist requires the expression of the GPR27 protein to record an increase in [lactate]_i_, the end product of aerobic glycolysis. Moreover, GPR27 is also involved in the regulation of resting levels of [lactate]_i_. We hope that this work will facilitate further studies into understanding the function of GPR27, which is highly expressed in the brain [3].

## Figures and Tables

**Table 1:** Responsiveness and integrated area under the curve (AUC) changes in FRET ratio signal upon exposure to stimuli in different experimental groups.

Agonist	% Responsive Cells (*n* = x/y), y = All Cells				Mean ± SEM AUC (%) All Cells (*n*)				Mean ± SEM AUC (%) Responsive Cells (*n*)			
	WT	KOBIE1	KOBIE1-FLAG	Astrocytes	WT	KOBIE1	KOBIE1-FLAG	Astrocytes	WT	KOBIE1	KOBIE1-FLAG	Astrocytes
8535	33.3 (*n* = 13/39)	0 (*n* = 0/20)	40.9 (*n* = 9/22)	59.2 (*n* = 42/71)	314.5 ± 90.7 (*n* = 39) ***^,#^	−50.9 ± 32.7 (*n* = 20)	464.2 ± 134.5 (*n* = 22) **	1515.4 ± 306.9 (*n* = 71) ***^,#^	855.2 ± 185.3 (*n* = 13) ***	–	931.7 ± 252.7 (*n* = 9) ***	3136.9 ± 283.5 (*n* = 42) ***^,###^
8535n	41.4 (*n* = 12/29)	0 (*n* = 0/17)	–	54.2 (*n* = 26/48)	396.9 ± 73.4 (*n* = 29) ***	−61.2 ± 37.8 (*n* = 17)	–	749.3 ± 193.4 (*n* = 48) ***	694.3 ± 126.6 (*n* = 12) ***	–	–	1330.4 ± 313.0 (*n* = 26) **
Control	0 (*n* = 0/24)	0 (*n* = 0/8)	0 (*n* = 0/13)	20.8 (*n* = 11/53)	−50.4 ± 32.9 (*n* = 24)	−34.6 ± 87.8 (*n* = 8)	−10.5 ± 65.6 (*n* = 13)	93.8 ± 42.3 (*n* = 53)	–	–	–	392.9 ± 55.4 (*n* = 11)

SEM, standard error of the mean; AUC, area under the curve; WT, wild-type; KOBIE1 and KOBIE1-FLAG, GPR27 knockout without and with GPR27-FLAG plasmid transfection, respectively. ** *p* ≤ 0.01; *** *p* ≤ 0.001 versus control cells. # *p* ≤ 0.05; ### *p* ≤ 0.001 versus 8535n.

## Data Availability

The data that support the findings of this study are available from the corresponding author upon reasonable request.

## Supplementary Materials

The following supporting information can be downloaded at: https://www.mdpi.com/article/10.3390/cells11061009/s1, Figure S1: Sequencing GPR27 knockout 3T3 cell line clones IA11, IE1 and IC5 as used in experiments. The deletions are marked by yellow lines. Figure S2: The HPLC purity of the GPR27 ligand 8535n.
