# Supplementary material for: The Activation of GPR27 Increases Cytosolic L-Lactate in 3T3 Embryonic Cells and Astrocytes
Source: Cells. 2022 Mar 16;11(6):1009. doi: 10.3390/cells11061009 (PMC8947442; doi:10.3390/cells11061009)
Supplement: Supplementary file 1 [file cells-11-01009-s001.zip › cells-1590687-supplementary.pdf]

Supplementary material

The Activation of GPR27 Increases Cytosolic L-Lactate in 3T3 Embryonic Cells and Astrocytes

Dorian Dolanc [1], Tomaž M. Zorec [2,3], Zala Smole [1], Anja Maver [1], Anemari Horvat [1,3], Thanigaimalai Pillaiyar [4], Saša Trkov Bobnar [3], Nina Vardjan [1,3], Marko Kreft [2,3,5], Helena Haque Chowdhury [1,3], and Robert Zorec [1,3,*]

[1] Laboratory of Neuroendocrinology, Molecular Cell Physiology, Institute of Pathophysiology, Faculty of Medicine, University of Ljubljana, 1000 Ljubljana, Slovenia; dorian.dolanc@mf.uni-lj.si (D.D.); zala.smole@mf.uni-lj.si (Z.S.); anja.maver97@gmail.com (A.M.); anemari.horvat@mf.uni-lj.si (A.H.); nina.vardjan@mf.uni-lj.si (N.V.); helena.chowdhury@celica.si (H.H.C.)

[2] Institute of Microbiology and Immunology, Faculty of Medicine, University of Ljubljana,
1000 Ljubljana, Slovenia; tomaz.zorec@celica.si (T.M.Z.); marko.kreft@celica.si (M.K.)

[3] Laboratory of Cell Engineering, Celica Biomedical, 1000 Ljubljana, Slovenia; sasa.trkov@gmail.com (S.T.B)

[4] Pharmaceutical/Medicinal Chemistry and Tübingen Center for Academic Drug Discovery, Institute of Pharmacy, Eberhard Karls University Tübingen, Auf der Morgenstelle 8, 72076 Tübingen, Germany; thanigaimalai.pillaiyar@uni-tuebingen.de

[5] Department of Biology, Biotechnical Faculty, University of Ljubljana, 1000 Ljubljana, Slovenia

* Correspondence: robert.zorec@mf.uni-lj.si (R.Z.).

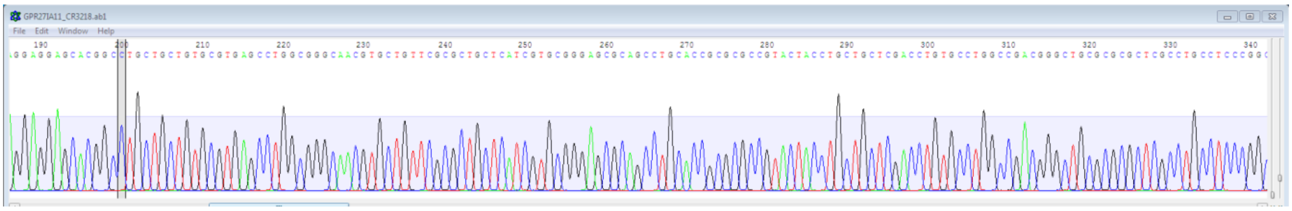

Figure S1. Sequencing GPR27 knockout 3T3 cell line clones IA11, IE1 and IC5 as used in experiments. The deletions are marked by yellow lines.

IA11: homozygous deletion -91

```
GPR27               CAGGGCGCAGGGAGTGGCCGTCGCCTCCTGCGCGCACCGCGACTGAGATGGCGGCGGCGG
mA                  CAGGGCGCAGGGAGTGGCCGTCGCCTCCTGCGCGCACCGCGACTGAGATGGCGGCGGCGG
mB                  CAGGGCGCAGGGAGTGGCCGTCGCCTCCTGCGCGCACCGCGACTGAGATGGCGGCGGCGG
mC                  CAGGGCGCAGGGAGTGGCCGTCGCCTCCTGCGCGCACCGCGACTGAGATGGCGGCGGCGG
                    ************************************************************

GPR27               CGGCGCGGCCGAGGCGCGCCGAGCCCCCGGGGCAGGGCCGGCCGGGCGCGGGCTGACAAC
mA                  CGGCGCGGCCGAGGCGCGCCGAGCCCCCGGGGCAGGGCCGGCCGGGCGCGGGCTGACAAC
mB                  CGGCGCGGCCGAGGCGCGCCGAGCCCCCGGGGCAGGGCCGGCCGGGCGCGGGCTGACAAC
mC                  CGGCGCGGCCGAGGCGCGCCGAGCCCCCGGGGCAGGGCCGGCCGGGCGCGGGCTGACAAC
                    ************************************************************

GPR27               CCCGCGGGCCGGGAGAGCTAGCGTGGAGGAGCGCAGGCCCCGGGGCGGCCTACGGCGAGC
mA                  CCCGCGGGCCGGGAGAGCTAGCGTGGAGGAGCGCAGGCCCCGGGGCGGCCTACGGCGAGC
mB                  CCCGCGGGCCGGGAGAGCTAGCGTGGAGGAGCGCAGGCCCCGGGGCGGCCTACGGCGAGC
mC                  CCCGCGGGCCGGGAGAGCTAGCGTGGAGGAGCGCAGGCCCCGGGGCGGCCTACGGCGAGC
                    ************************************************************
                                                                      g1471
GPR27               GGCGAGGGCCGGCGGGGAGGAGGAGGAGCACGGCCGCGATGGCGAACGCTAGTGAGCCGG
mA                  GGCGAGGGCCGGTGGGGAGGAGGAGGAGCACG---------------------------- -91
mB                  GGCGAGGGCCGGTGGGGAGGAGGAGGAGCACG---------------------------- -91
mC                  GGCGAGGGCCGGTGGGGAGGAGGAGGAGCACG---------------------------- -91
                    ************ *******************

GPR27               GCGGCGGCGGCAGCGGCGGAGGGGCCGAGGCTGCCGCGCTGGGCCTTAGGCTGGCCACGC
mA                  ------------------------------------------------------------
mB                  ------------------------------------------------------------
mC                  ------------------------------------------------------------

GPR27               TCAGCCTGCTGCTGTGCGTGAGCCTGGCGGGCAACGTGCTGTTCGCGCTGCTCATCGTGC
mA                  ---GCCTGCTGCTGTGCGTGAGCCTGGCGGGCAACGTGCTGTTCGCGCTGCTCATCGTGC
mB                  ---GCCTGCTGCTGTGCGTGAGCCTGGCGGGCAACGTGCTGTTCGCGCTGCTCATCGTGC
mC                  ---GCCTGCTGCTGTGCGTGAGCCTGGCGGGCAACGTGCTGTTCGCGCTGCTCATCGTGC
                       *********************************************************

GPR27               GGGAGCGCAGCCTGCACCGCGCGCCGTACTACCTGCTGCTCGACCTGTGCCTGGCCGACG
mA                  GGGAGCGCAGCCTGCACCGCGCGCCGTACTACCTGCTGCTCGACCTGTGCCTGGCCGACG
mB                  GGGAGCGCAGCCTGCACCGCGCGCCGTACTACCTGCTGCTCGACCTGTGCCTGGCCGACG
mC                  GGGAGCGCAGCCTGCACCGCGCGCCGTACTACCTGCTGCTCGACCTGTGCCTGGCCGACG
                    ************************************************************

GPR27               GGCTGCGCGCGCTCGCCTGCCTCCCGGCCGTCATGCTGGCGGCGCGGCGCGCGGCGGCCG
mA                  GGCTGCGCGCGCTCGCCTGCCTCCCGGCCGTCATGCTGGCGGCGCGGCGCGCGGCGGCCG
mB                  GGCTGCGCGCGCTCGCCTGCCTCCCGGCCGTCATGCTGGCGGCGCGGCGCGCGGCGGCCG
mC                  GGCTGCGCGCGCTCGCCTGCCTCCCGGCCGTCATGCTGGCGGCGCGGCGCGCGGCGGCCG
                    ************************************************************

GPR27               CGGCGGGGACGCCGCCGGGCGCGCTGGGCTGCAAGCTGCTGGCCTTCCTGGCCGCACTCT
mA                  CGGCGGGGACGCCGCCGGGCGCGCTGGGCTGCAAGCTGCTGGCCTTCCTGGCCGCACTCT
mB                  CGGCGGGGACGCCGCCGGGCGCGCTGGGCTGCAAGCTGCTGGCCTTCCTGGCCGCACTCT
mC                  CGGCGGGGACGCCGCCGGGCGCGCTGGGCTGCAAGCTGCTGGCCTTCCTGGCCGCACTCT
                    ************************************************************
```

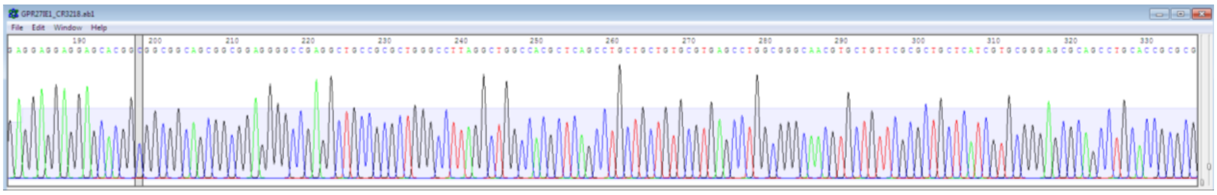

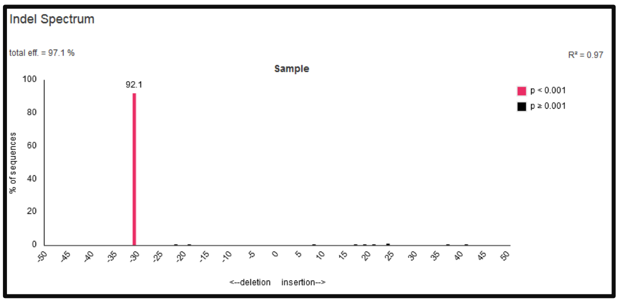

IE1: homozygous deletion -31

```
GPR27          CCAGGGCGCAGGGAGTGGCCGTCGCCTCCTGCGCGCACCGCGACTGAGATGGCGGCGGCG
mB             CCAGGGCGCAGGGAGTGGCCGTCGCCTCCTGCGCGCACCGCGACTGAGATGGCGGCGGCG
mF             CCAGGGCGCAGGGAGTGGCCGTCGCCTCCTGCGCGCACCGCGACTGAGATGGCGGCGGCG
mG             CCAGGGCGCAGGGAGTGGCCGTCGCCTCCTGCGCGCACCGCGACTGAGATGGCGGCGGCG
mK             CCAGGGCGCAGGGAGTGGCCGTCGCCTCCTGCGCGCACCGCGACTGAGATGGCGGCGGCG
               ************************************************************

GPR27          GCGGCGCGGCCGAGGCGCGCCGAGCCCCCGGGGCAGGGCCGGCCGGGCGCGGGCTGACAA
mB             GCGGCGCGGCCGAGGCGCGCCGAGCCCCCGGGGCAGGGCCGGCCGGGCGCGGGCTGACAA
mF             GCGGCGCGGCCGAGGCGCGCCGAGCCCCCGGGGCAGGGCCGGCCGGGCGCGGGCTGACAA
mG             GCGGCGCGGCCGAGGCGCGCCGAGCCCCCGGGGCAGGGCCGGCCGGGCGCGGGCTGACAA
mK             GCGGCGCGGCCGAGGCGCGCCGAGCCCCCGGGGCAGGGCCGGCCGGGCGCGGGCTGACAA
               ************************************************************

GPR27          CCCCGCGGGCCGGGAGAGCTAGCGTGGAGGAGCGCAGGCCCCGGGGCGGCCTACGGCGAG
mB             CCCCGCGGGCCGGGAGAGCTAGCGTGGAGGAGCGCAGGCCCCGGGGCGGCCTACGGCGAG
mF             CCCCGCGGGCCGGGAGAGCTAGCGTGGAGGAGCGCAGGCCCCGGGGCGGCCTACGGCGAG
mG             CCCCGCGGGCCGGGAGAGCTAGCGTGGAGGAGCGCAGGCCCCGGGGCGGCCTACGGCGAG
mK             CCCCGCGGGCCGGGAGAGCTAGCGTGGAGGAGCGCAGGCCCCGGGGCGGCCTACGGCGAG
               ************************************************************
                                                                 g1471
GPR27          CGGCGAGGGCCGGCGGGGAGGAGGAGGAGCACGGCCGCGATGGCGAACGCTAGTGAGCCG
mB             CGGCGAGGGCCGGCGGGGAGGAGGAGGAGCA----------------------------- -31
mF             CGGCGAGGGCCGGCGGGGAGGAGGAGGAGCA----------------------------- -31
mG             CGGCGAGGGCCGGCGGGGAGGAGGAGGAGCA----------------------------- -31
mK             CGGCGAGGGCCGGCGGGGAGGAGGAGGAGCA----------------------------- -31
               *******************************

GPR27          GGCGGCGGCGGCAGCGGCGGAGGGGCCGAGGCTGCCGCGCTGGGCCTTAGGCTGGCCACG
mB             --CGGCGGCGGCAGCGGCGGAGGGGCCGAGGCTGCCGCGCTGGGCCTTAGGCTGGCCACG
mF             --CGGCGGCGGCAGCGGCGGAGGGGCCGAGGCTGCCGCGCTGGGCCTTAGGCTGGCCACG
mG             --CGGCGGCGGCAGCGGCGGAGGGGCCGAGGCTGCCGCGCTGGGCCTTAGGCTGGCCACG
mK             --CGGCGGCGGCAGCGGCGGAGGGGCCGAGGCTGCCGCGCTGGGCCTTAGGCTGGCCACG
                 **********************************************************

GPR27          CTCAGCCTGCTGCTGTGCGTGAGCCTGGCGGGCAACGTGCTGTTCGCGCTGCTCATCGTG
mB             CTCAGCCTGCTGCTGTGCGTGAGCCTGGCGGGCAACGTGCTGTTCGCGCTGCTCATCGTG
mF             CTCAGCCTGCTGCTGTGCGTGAGCCTGGCGGGCAACGTGCTGTTCGCGCTGCTCATCGTG
mG             CTCAGCCTGCTGCTGTGCGTGAGCCTGGCGGGCAACGTGCTGTTCGCGCTGCTCATCGTG
mK             CTCAGCCTGCTGCTGTGCGTGAGCCTGGCGGGCAACGTGCTGTTCGCGCTGCTCATCGTG
               ************************************************************

GPR27          CGGGAGCGCAGCCTGCACCGCGCGCCGTACTACCTGCTGCTCGACCTGTGCCTGGCCGAC
mB             CGGGAGCGCAGCCTGCACCGCGCGCCGTACTACCTGCTGCTCGACCTGTGCCTGGCCGAC
mF             CGGGAGCGCAGCCTGCACCGCGCGCCGTACTACCTGCTGCTCGACCTGTGCCTGGCCGAC
mG             CGGGAGCGCAGCCTGCACCGCGCGCCGTACTACCTGCTGCTCGACCTGTGCCTGGCCGAC
mK             CGGGAGCGCAGCCTGCACCGCGCGCCGTACTACCTGCTGCTCGACCTGTGCCTGGCCGAC
               ************************************************************
```

```
GPR27          GGGCTGCGCGCGCTCGCCTGCCTCCCGGCCGTCATGCTGGCGGCGCGGCGCGCGGCGGCC
mB             GGGCTGCGCGCGCTCGCCTGCCTCCCGGCCGTCATGCTGGCGGCGCGGCGCGCGGCGGCC
mF             GGGCTGCGCGCGCTCGCCTGCCTCCCGGCCGTCATGCTGGCGGCGCGGCGCGCGGCGGCC
mG             GGGCTGCGCGCGCTCGCCTGCCTCCCGGCCGTCATGCTGGCGGCGCGGCGCGCGGCGGCC
mK             GGGCTGCGCGCGCTCGCCTGCCTCCCGGCCGTCATGCTGGCGGCGCGGCGCGCGGCGGCC
               ************************************************************
```

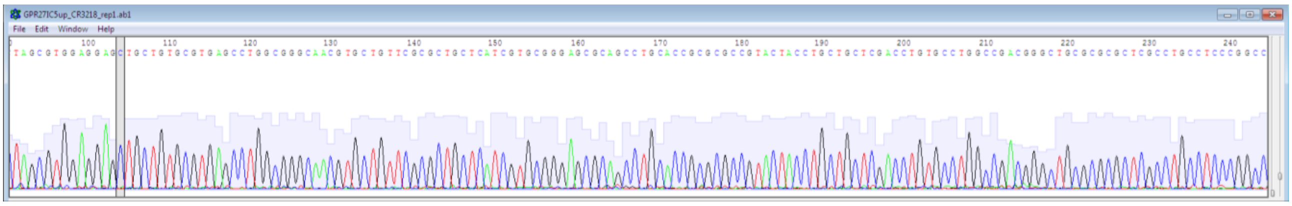

IC5 heterozygous deletion: -316 (lower band) / -157 (upper band)

IC5 -316 (lower band)

```
GPR27          CCAGCCCAGTCAGGCCCGGTGCGGCGCGGGATGCCTCGGCGGGACTGGCGGCGGCGGCCA
mB             CCAGCCCAGTCAGGCCCGGTGCGGCGCGGGATGCCTCGGCGGGACTGGCGGCGGCGGCCA
mE             CCAGCCCAGTCAGGCCCGGTGCGGCGCGGGATGCCTCGGCGGGACTGGCGGCGGCGGCCA
mL             CCAGCCCAGTCAGGCCCGGTGCGGCGCGGGATGCCTCGGCGGGACTGGCGGCGGCGGCCA
               ************************************************************

GPR27          CGCTGCGCGCTCCGGCGGAGGGGAGGGGGCTCGGCGGCGGCGGCGAAGGCGGTGGCGAAG
mB             CGCTGCGCGCTCCGGCGGAGGGGAGGGGGCTCGGCGGCGGCGGCGAAGGCGGTGGCGAAG
mE             CGCTGCGCGCTCCGGCGGAGGGGAGGGGGCTCGGCGGCGGCGGCGAAGGCGGTGGCGAAG
mL             CGCTGCGCGCTCCGGCGGAGGGGAGGGGGCTCGGCGGCGGCGGCGAAGGCGGTGGCGAAG
               ************************************************************

GPR27          GCGGCGGCAGCGGCAGCGGCAGCGGCGGGCCCGGCGGGGGGCAGAGCCAAGAGGCGCGGC
mB             GCGGCGGCAGCGGCAGCGGCAGCGGCGGGCCCGGCGGGGGGCA-----------------
mE             GCGGCGGCAGCGGCAGCGGCAGCGGCGGGCCCGGCGGGGGGCA-----------------
mL             GCGGCGGCAGCGGCAGCGGCAGCGGCGGGCCCGGCGGGGGGCA-----------------
               *******************************************

GPR27          GGCGGAGGAGGAACGGGAGGAGGACGCGGAGCATGCGGGGGGCTGCAGGCCTGCCCGCAG
mB             --------------------------------------------------------GCAG
mE             --------------------------------------------------------GCAG
mL             --------------------------------------------------------GCAG
                                                                       ****

GPR27          TGAAGCCCGCCGCGCCCAGGGCGCAGGGAGTGGCCGTCGCCTCCTGCGCGCACCGCGACT
mB             GGAAAC------------------------------------------------------
mE             GGAAAC------------------------------------------------------
mL             GGAAAC------------------------------------------------------
                *** *

GPR27          GAGATGGCGGCGGCGGCGGCGCGGCCGAGGCGCGCCGAGCCCCCGGGGCAGGGCCGGCCG
mB             ------------------------------------------------------------
mE             ------------------------------------------------------------
mL             ------------------------------------------------------------

GPR27          GGCGCGGGCTGACAACCCCGCGGGCCGGGAGAGCTAGCGTGGAGGAGCGCAGGCCCCGGG
mB             --------------------TGGGCTCGGAAA-----------------CAGTCCCT---
mE             --------------------TGGGCTCGGAAA-----------------CAGTCCCT---
mL             --------------------TGGGCTCGGAAA-----------------CAGTCCCT---
                                    ****  *** *                 *** ***

GPR27          GCGGCCTACGGCGAGCGGCGAGGGCCGGCGGGGAGGAGGAGGAGCACGGCCGCGATGGCG
mB             ------------------------------------------------------------
mE             ------------------------------------------------------------
mL             ------------------------------------------------------------

                     g1471
GPR27          AACGCTAGTGAGCCGGGCGGCGGCGGCAGCGGCGGAGGGGCCGAGGCTGCCGCGCTGGGC
mB             -----------------------------CGGCGGAGGGGCCGAGGCTGCCGCGCTGGGC  -316
```

```
mE                  -----------------------------CGGCGGAGGGGCCGAGGCTGCCGCGCTGGGC -316
mL                  -----------------------------CGGCGGAGGGGCCGAGGCTGCCGCGCTGGGC -316
                                                 *******************************

GPR27               CTTAGGCTGGCCACGCTCAGCCTGCTGCTGTGCGTGAGCCTGGCGGGCAACGTGCTGTTC
mB                  CTTAGGCTGGCCACGCTCAGCCTGCTGCTGTGCGTGAGCCTGGCGGGCAACGTGCTGTTC
mE                  CTTAGGCTGGCCACGCTCAGCCTGCTGCTGTGCGTGAGCCTGGCGGGCAACGTGCTGTTC
mL                  CTTAGGCTGGCCACGCTCAGCCTGCTGCTGTGCGTGAGCCTGGCGAGCAACGTGCTGTTC
                    ********************************************* **************

GPR27               GCGCTGCTCATCGTGCGGGAGCGCAGCCTGCACCGCGCGCCGTACTACCTGCTGCTCGAC
mB                  GCGCTGCTCATCGTGCGGGAGCGCAGCCTGCACCGCGCGCCGTACTACCTGCTGCTCGAC
mE                  GCGCTGCTCATCGTGCGGGAGCGCAGCCTGCACCGCGCGCCGTACTACCTGCTGCTCGAC
mL                  GCGCTGCTCATCGTGCGGGAGCGCAGCCTGCACCGCGCGCCGTACTACCTGCTGCTCGAC
                    ************************************************************
```

IC5 -157 (upper band)

```
GPR27               CCGCGCCCAGGGCGCAGGGAGTGGCCGTCGCCTCCTGCGCGCACCGCGACTGAGATGGCG
mA                  CCGCGCCCAGGGCGCAGGGAGTGGCCGTCGCCTCCTGCGCGCACCGCGACTGAGATGGCG
mK                  CCGCGCCCAGGGCGCAGGGAGTGGCCGTCGCCTCCTGCGCGCACCGCGACTGAGATGGCG
                    ************************************************************

GPR27               GCGGCGGCGGCGCGGCCGAGGCGCGCCGAGCCCCCGGGGCAGGGCCGGCCGGGCGCGGGC
mA                  GCGGCGGCGGCGCGGCCGAGGCGCGCCGAGCCCCCGGGGCAGGGCCGGCCGGGCGCGGGC
mK                  GCGGCGGCGGCGCGGCCGAGGCGCGCCGAGCCCCCGGGGCAGGGCCGGCCGGGCGCGGGC
                    ************************************************************

GPR27               TGACAACCCCGCGGGCCGGGAGAGCTAGCGTGGAGGAGCGCAGGCCCCGGGGCGGCCTAC
mA                  TGACAACCCCGCGGGCCGGGAGAGCTAGCGTGGAGGA-----------------------
mK                  TGACAACCCCGCGGGCCGGGAGAGCTAGCGTGGAAGA-----------------------
                    ********************************** **
                                                                   g1471
GPR27               GGCGAGCGGCGAGGGCCGGCGGGGAGGAGGAGGAGCACGGCCGCGATGGCGAACGCTAGT
mA                  ------------------------------------------------------------ -157
mK                  ------------------------------------------------------------ -157

GPR27               GAGCCGGGCGGCGGCGGCAGCGGCGGAGGGGCCGAGGCTGCCGCGCTGGGCCTTAGGCTG
mA                  ------------------------------------------------------------
mK                  ------------------------------------------------------------

GPR27               GCCACGCTCAGCCTGCTGCTGTGCGTGAGCCTGGCGGGCAACGTGCTGTTCGCGCTGCTC
mA                  --------------CCTGCTGTGCGTGAGCCTGGCGGGCAACGTGCTGTTCGCGCTGCTC
mK                  --------------GCTGCTGTGCGTGAGCCTGGCGGGCAACGTGCTGTTCGCGCTGCTC
                                   *********************************************
```

Figure S2. The HPLC purity of the GPR27 ligand 8535n. The purity of the test compound was determined via reverse phase high performance liquid chromatography (RP-HPLC) using Phenomenex Luna C8 RP columns (150 × 4.6 mm, 5 μm) on an Agilent 1100 Series LC with UV diode array detector (DAD) at λ = 254 and 230 nm.

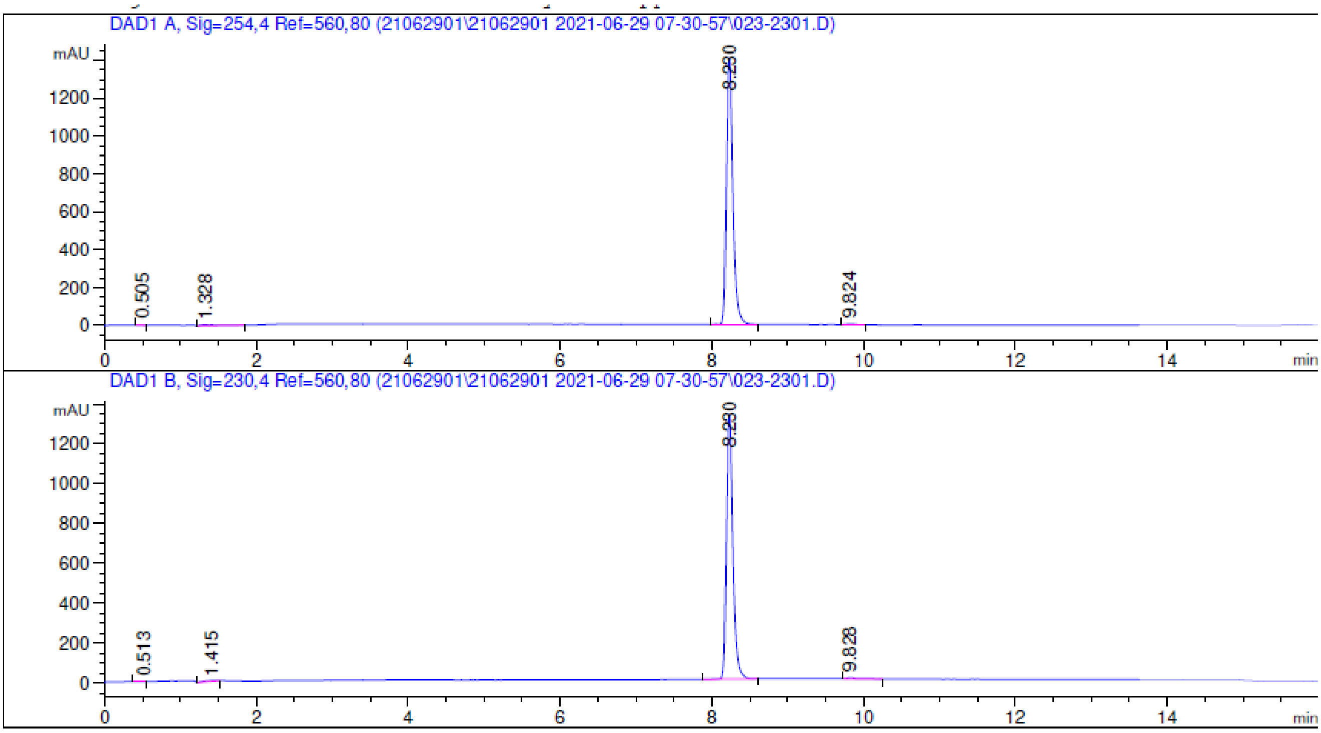

Signal 1: DAD1 A, Sig=254,4 Ref=560,80

Peak #	RetTime [min]	Type	Width [min]	Area [mAU*s]	Height [mAU]	Area %
1	0.505	BB	0.0572	8.27236	2.13463	0.0992
2	1.328	BB	0.2084	115.46124	6.78895	1.3845
3	8.230	BB	0.0875	8189.43213	1415.10449	98.2012
4	9.824	BB	0.0922	26.27888	4.24491	0.3151

Signal 2: DAD1 B, Sig=230,4 Ref=560,80

Peak #	RetTime [min]	Type	Width [min]	Area [mAU*s]	Height [mAU]	Area %
1	0.513	BB	0.0684	7.38316	1.52805	0.0937
2	1.415	BB	0.1686	57.50241	4.45341	0.7298
3	8.230	BB	0.0879	7773.30273	1334.94006	98.6550
4	9.828	BB	0.1060	41.08839	5.44636	0.5215
